# RNA polymerase III transcription and cancer: A tale of two RPC7 subunits

**DOI:** 10.3389/fmolb.2022.1073795

**Published:** 2023-01-12

**Authors:** Ruiying Cheng, Kevin Van Bortle

**Affiliations:** ^1^ Department of Cell and Developmental Biology, University of Illinois Urbana-Champaign, Urbana, IL, United States; ^2^ Cancer Center at Illinois, University of Illinois Urbana-Champaign, Urbana, IL, United States

**Keywords:** Pol III and cancer, *POLR3G* and *POLR3GL*, RPC7α and RPC7β, RPC7, RPC32, tRNA

## Abstract

RNA polymerase III composition is shaped by the mutually exclusive incorporation of two paralogous subunits, RPC7α and RPC7β, encoded by genes *POLR3G* and *POLR3GL* in vertebrates. The expression of *POLR3G* and *POLR3GL* is spatiotemporally regulated during development, and multiple reports point to RPC7α-enhanced Pol III activity patterns, indicating that Pol III identity may underly dynamic Pol III transcription patterns observed in higher eukaryotes. In cancer, upregulation of *POLR3G*, but not *POLR3GL*, is associated with poor survival outcomes among patients, suggesting differences between RPC7α and RPC7β further influence disease progression and may translate into future biomarkers and therapeutic strategies. Here, we outline our current understanding of Pol III identity and transcription and reexamine the distinct protein characteristics of Pol III subunits RPC7α and RPC7β. Drawing on both structural and genomic studies, we discuss differences between RPC7α and RPC7β and the potential mechanisms by which Pol III identity may establish differential activities during development and disease.

## Introduction

The RNA polymerase III (Pol III) machinery produces multiple classes of small non-coding RNA (ncRNA) with integral roles in translation, transcription regulation, RNA processing, and other fundamental processes. In humans, the Pol III transcriptome includes tRNA and 5S rRNA, 7SK, U6, and U6atac small nuclear RNA (snRNA), RNase P/MRP catalytic RNAs RPPH1 (H1) and RMRP, 7SL and 7SL-derived Alu, BC200, and snaR RNA, vault RNA, Y RNA, and nc886 (Dieci et al., 2013). Beyond the core functions established for each small ncRNA subclass, the Pol III transcriptome can drive endogenous immune signaling pathways through the activation of pattern recognition receptors, sequester specific proteins and miRNAs in ways that shape cell growth and proliferation, and modulate the expression and behavior of oncogenes and tumor suppressor genes (Kessler and Maraia, 2021). In this way, Pol III transcription and Pol III-derived small ncRNA are understood to play important roles in cancer and disease (Yeganeh and Hernandez, 2020). Pol III activity is controlled through multiple regulatory layers and mechanisms that intersect extracellular growth cues and, as we describe in this review, pathways that can modulate Pol III subunit composition and drive downstream changes in Pol III transcription.

**FIGURE 1:**
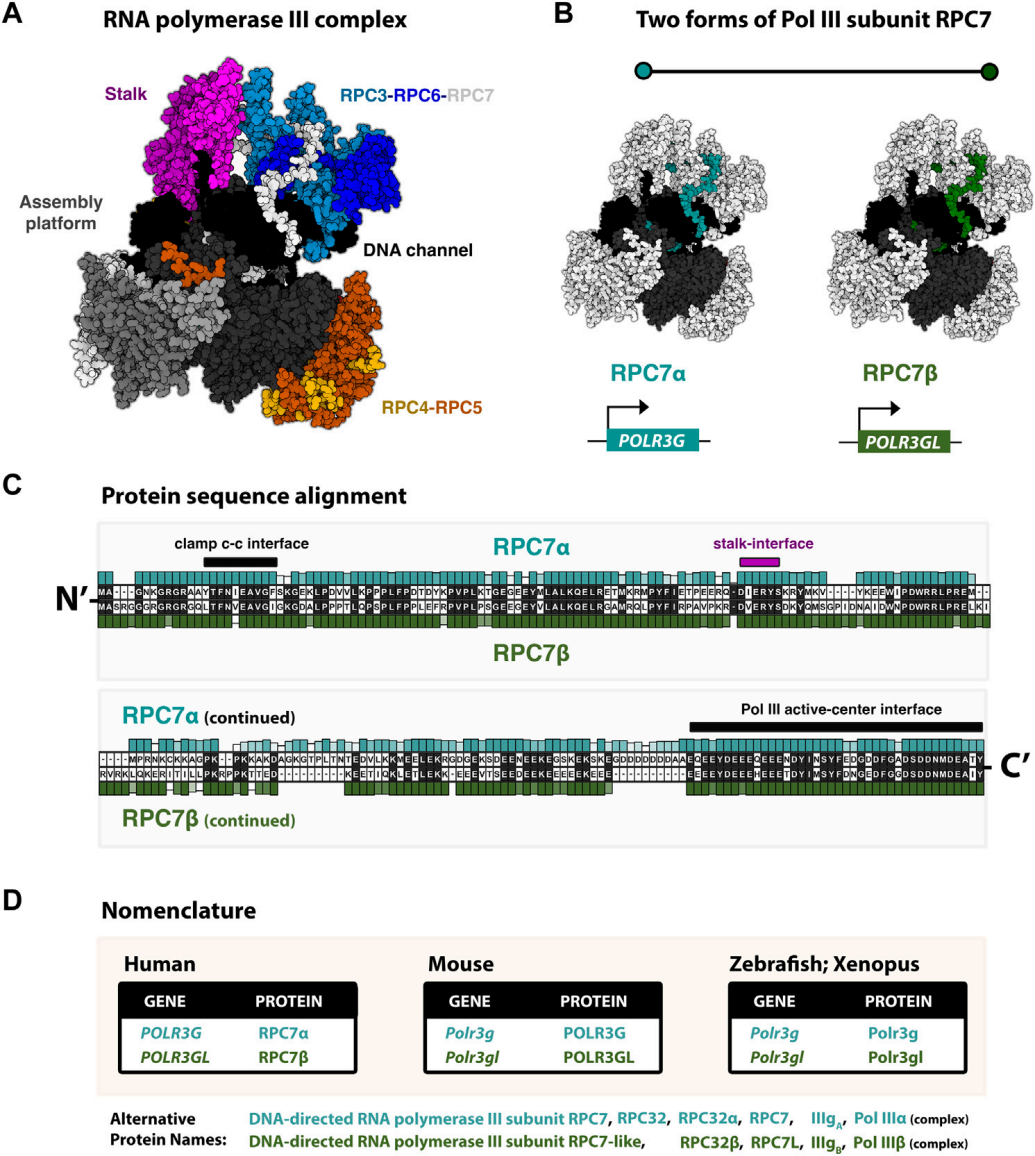
RNA polymerase III identity: paralogous Pol III subunits RPC7α and RC7β (*POLR3G* and *POLR3GL*) **(A)** Cryo-EM structure (7D59, Wang et al., 2021) of the human RNA polymerase III complex. Structure re-colored in Mol* to highlight major structural modules, including the stably associated RPC3-RPC6-RPC7 subcomplex. **(B)** Pol III structure with emphasis on RPC7 subunits (blue/green), which are highlighted for generalization. True structure corresponds to subunit RPC7α. Human subunits RPC7α and RPC7β are encoded by genes *POLR3G* and *POLR3GL*, respectively. **(C)** Protein sequence alignment of RPC7 subunits. Human RPC7α and RPC7β primary amino acid sequences are shown, as well as their respective conservation levels across other forms of either mammalian subunit. **(D)** Table summary of the current species-specific gene and protein nomenclatures for paralogous RPC7 subunits in Human, Mouse, Zebrafish, and Xenopus.

Structurally, Pol III is composed of 17 subunits, including 12 core subunits and five additional subunits that assemble into two Pol III-specific subcomplexes, RPC3-RPC6-RPC7 and RPC4-RPC5, involved in transcription initiation, elongation, and termination (Vannini and Cramer, 2012) (Figure 1A). Among the core subunits, five are shared between Pols I, II, and III, and two more subunits are shared between Pols I and III (Geiduschek and Kassavetis, 2001). The Pol III-specific heterotrimer RPC3-RPC6-RPC7 and heterodimer RPC3-RPC4 are partially equivalent to the Pol II general transcription factors, TFIIE and TFIIF, respectively (Hoffmann et al., 2016). Changes in Pol III subunit composition occur within the ternary “TFIIE-like” subcomplex composed of RPC3, RPC6, and RPC7, which is required for initiation at Pol III promoters but is otherwise dispensable for transcription elongation *in vitro* (Wang and Roeder, 1997). Specifically, subunit RPC7 is encoded by two paralogous genes, *POLR3G* and *POLR3GL*, which are developmentally regulated and produce similar but non-identical proteins RPC7α and RPC7β, the only mammalian RNA polymerase III subunit variants identified to date (Figure 1B). In this review, we outline our current understanding of the regulation and function of RPC7α and RPC7β, beginning with the discoveries and initial characterizations of both subunits. By examining the reported similarities and differences in RPC7 subunit sequence and activities during development and disease, we seek to re-visit the question: are RPC7α and RPC7β subunits functionally redundant?

## The heterogeneity of RNA polymerase III: Two Pol III isoforms

Two forms of Pol III, termed III_A_ and III_B_, were first biochemically purified from mouse myeloma tumor cells and shown to have similar sensitivities to ammonium sulfate and α-amanitin (Schwartz et al., 1974). Pol III_A_ and III_B_ were subsequently found to have similar catalytic properties and to be largely identical in composition, with the exception of a single subunit at molecular weight 32 kDa (“IIIg_A_”), which is replaced with an alternate 33 kDa protein (“IIIg_B_”) (Sklar and Roeder, 1976). The initial characterizations of III_A_ and III_B_ by Robert (Bob) Roeder and colleagues also identified comparative enrichment of Pol III_B_ within the cytoplasmic fractions (Schwartz et al., 1974). However, with no additional follow-up on III_A_ and III_B_, the significance of differential Pol III subunit composition remained unexplored for several decades until the discovery of two human Pol III isoforms, termed Pol III_α_ and Pol III_β_, also delineated by similar but non-identical proteins of approximately 32 kDa (Haurie et al., 2010). The two subunits, RPC32α and RPC32β, were shown to be predominantly nuclear in human HeLa cells and to be assembled into Pol III complexes that otherwise share the same subunit composition as first reported for III_A_ and III_B_.

As we now understand, the paralogous RPC32α and RPC32β proteins function as highly similar components of the ternary RPC3-RPC6-RPC7 subcomplex and are hereafter referred to as RPC7α and RPC7β. Comparative sequence analysis of the genes encoding RPC7α and RPC7β, *POLR3G* and *POLR3GL*, suggest gene duplication events gave rise to the two forms of RPC7 with origins in a common ancestor of vertebrates (Renaud et al., 2014). Ensuing functional experiments demonstrated that ectopic expression of *POLR3G*, which is otherwise more highly expressed in immortalized and dividing cells, enhances colony formation, upregulates specific Pol III-transcribed genes, and promotes expression of transformation-associated genes, suggesting non-equivalent properties for RPC7α and RPC7β (Haurie et al., 2010; Renaud et al., 2014). Initial genome-wide mapping of RPC7α and RPC7β uncovered highly similar localization patterns, however, suggesting subunit incorporation does not directly modulate gene target specificity (Renaud et al., 2014). Instead, differences in Pol III identity, determined by the presence of RPC7α or RPC7β, are likely to influence Pol III transcription and cell transformation through alternate mechanisms that we explore in this review.

## The RPC3-RPC6-RPC7 subcomplex

RPC7α and RPC7β are mutually exclusive components of the RPC3-RPC6-RPC7 heterotrimer and are thought to analogously function as a molecular “stalk bridge.” A specific, highly conserved region within RPC7 extrudes from the RPC3-RPC6-RPC7 complex and closely interacts with the Pol III stalk module (Girbig et al., 2021). The stalk bridge ostensibly functions in conformational changes in Pol III structure during transitions between apo- to transcription-states, and deletion of the RPC7 stalk bridge interface motif is lethal in *S. cerevisiae*, suggesting an essential role in this process (Wang et al., 2021). The stalk module, which is composed of subunits RPC8 and RPC9, engages in transcription initiation and interactions with nascent small ncRNA during transcription (Jasiak et al., 2006). The RPC3-RPC6-RPC7 subcomplex also functions in transcription initiation through direct interactions between RPC6 and transcription factor complex TFIIIB subunits at Pol III target genes (Wang and Roeder, 1997; Kenneth et al., 2008). However, the core Pol III complex, absent RPC3-RPC6-RPC7, is capable of transcription elongation and termination, suggesting the necessity of the RPC3-RPC6-RPC7 subcomplex is limited to the earliest stages of recruitment and transcription initiation at Pol III target genes (Wang and Roeder, 1997). How, then, can RPC7α and RPC7β differentially modulate the activity of RNA polymerase III? Recent structural investigations point to multiple putative mechanisms on the basis of differences in protein sequence and mapped Pol III protein interfaces.

## Divergent protein sequence and implications for RPC7α and RPC7β function

In humans, *POLR3G*, located on chromosome 5, and *POLR3GL*, located on chromosome 1, share similar exon/intron structure, suggesting the evolution of multiple vertebrate RPC7 proteins arose through an ancestral gene duplication event (Renaud et al., 2014). RPC7β is more similar, according to protein sequence alignment, to RPC7 subunits in non-vertebrate species (Renaud et al., 2014). In addition to *POLR3G* and *POLR3GL*, two annotated pseudogenes, located on chromosomes 14 (*POLR3GP1*) and 18 (*POLR3GP2*), are characterized by sequences mapping to mRNA transcripts that encode RPC7α. The genetic copies of multiple spliced *POLR3G* transcripts imply subsequent retroduplication events have given rise to additional forms of *POLR3G*, though any evidence of *POLR3GP1* and *POLR3GP2* expression is currently lacking.

**FIGURE 2:**
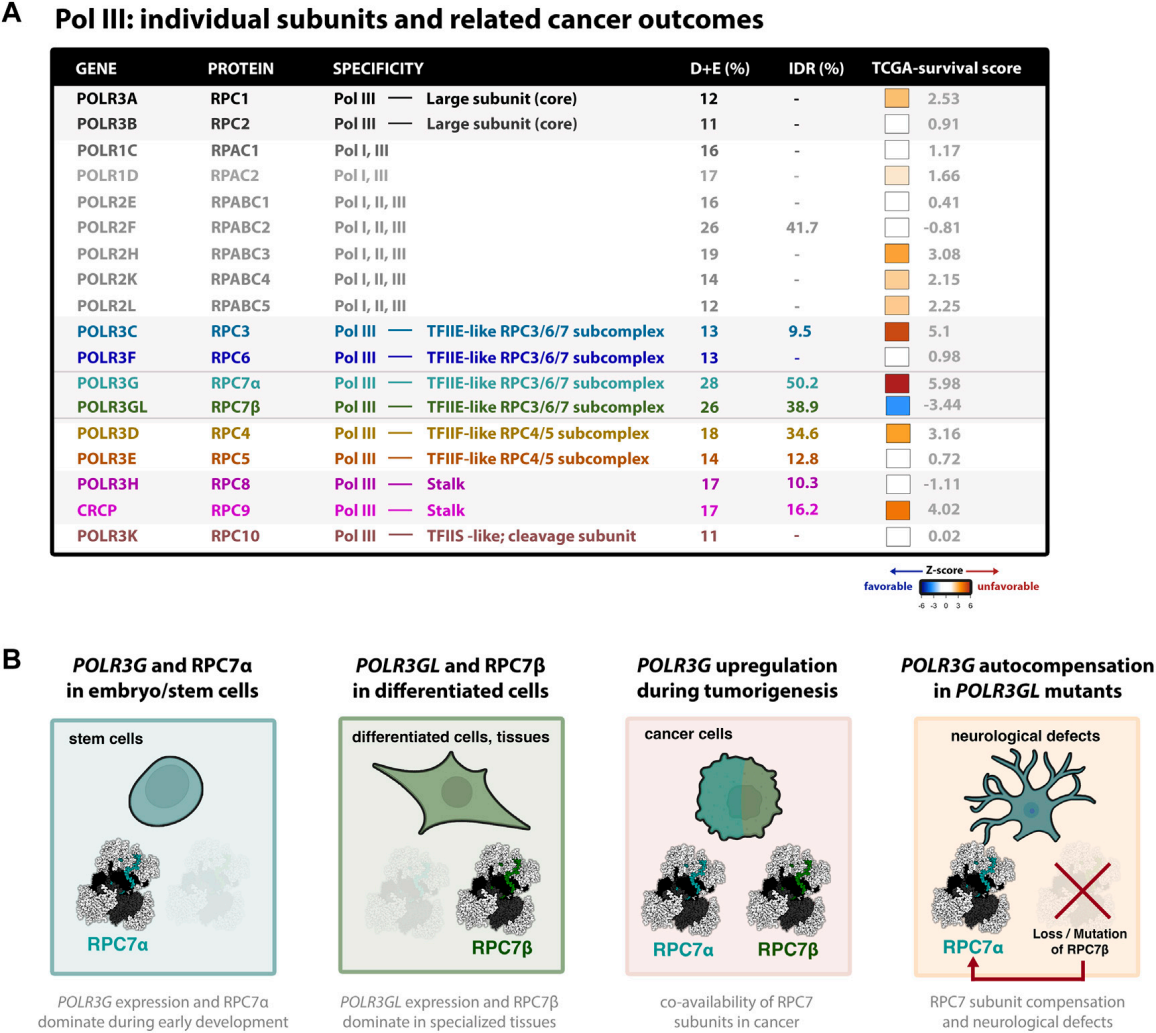
Dynamic expression and clinical outcome signatures of the cancer-associated Pol III subunit RPC7α **(A)** Table summary of all human Pol III subunits and basic features; colors correspond to cryo-EM structure shown in Figure 1A. The association of each subunit and cancer outcomes, which connects gene expression (RNA-seq) with pan-cancer survival statistics, is represented by z-score (tcga-survival.com). High Z-scores (red) are associated with unfavorable cancer outcomes, whereas low z-scores (blue) are associated with favorable outcomes. Survival analysis was acquired from tcga-survival.com version 2.0, which includes TCGA outcome data from 10,884 patients and 33 cancer types (Smith and Sheltzer, 2022). **(B)** Illustrations of Pol III developmental regulation, such that *POLR3G* expression and RPC7α availability peaks during early development, in contrast to *POLR3GL* expression and RPC7β availability observed in differentiated cells and specialized tissues. Also shown are illustrations that *POLR3G* upregulation results in co-availability of RPC7α and RPC7β in cancer cells, resulting in enhanced Pol III transcription activity, and that mutation and functional loss of RPC7β requires autocompensation by *POLR3G* expression and RPC7α availability in differentiated cells and tissues. Evidence suggests that RPC7α rescue may result in neurological defects and, in mice, premature death. Cell models were created with BioRender.com.

The human forms of RPC7α and RPC7β share 46% amino acid identity (Renaud et al., 2014). The protein sequence alignment of human RPC7α and RPC7β is shown in Figure 1C, and the corresponding nomenclature of homologous genes and proteins reported in Mouse, Zebrafish, and Xenopus studies are provided in Figure 1D. Pairwise alignment of RPC7α and RPC7β shows that differences in amino acid sequence are distributed throughout the primary sequences for each protein and indicate a high degree of similarity within notable structural interfaces (Figure 1C). Both RPC7α and RPC7β feature high densities of negatively charged amino acids (28% and 26% D+E, respectively), and include predicted intrinsically disordered regions (IDRs) in both N- and C-termini. However, compared to RPC7β, the C-terminus of RPC7α includes a unique run of aspartic acid residues and, overall, RPC7α is the most highly disordered protein among Pol III subunits (Figure 2A). Additional differences include particularly divergent sequence features within central regions that are otherwise highly conserved among RPC7α or RPC7β across mammals, suggesting potentially important roles in distinguishing RPC7α and RPC7β function.

Though direct structural comparisons of the Pol III complex assembled with RPC7α or RPC7β are currently lacking, hypothetical mechanistic models of differential RPC7α and RPC7β function can be predicted through integration of mapped protein-protein interfaces with the divergent sequence features of RPC7 subunits. For example, Cryo-EM structures of the human Pol III complex pinpoint specific interactions between the N-terminus of RPC7 with the Pol III clamp domain that, through unique aromatic stacking interactions and hydrogen bond formation, is predicted to be stronger for RPC7α than for RPC7β (Girbig et al., 2021). The RPC7-clamp-binding site overlaps reported docking sites between Pol III and the transcriptional repressor Maf1 in *S. cerevisiae* (Vorländer et al., 2020), suggesting the N-termini of RPC7α and RPC7β may differentially preclude MAF1-mediated repression of Pol III (Girbig et al., 2021). The C-termini of RPC7α and RPC7β, which are highly conserved across RPC7 proteins (Figure 1C), are also of particular interest, as Cryo-EM structures of human Pol III unexpectedly discovered insertion of the RPC7α C-terminus into the active center of Pol III, directly occupying the DNA-binding site and extending to the RNA exit channel (Wang et al., 2021). C-terminal deletion of the RPC7 ortholog in yeast, as well as alanine substitution at residues occupying the active center cause severe growth defects or death, suggesting an essential role in Pol III function (Thuillier et al., 1995; Wang et al., 2021). Current models propose interactions between RPC7 and TFIIIB subunit BRF1 may restructure the RPC7 C-terminal tail to facilitate Pol III transcription initiation (Wang et al., 2022). Further deconstructing the role of the N- and C-termini of RPC7 subunits is critical for understanding the functions and putative differences between RPC7α or RPC7β in Pol III transcription and regulation.

## Dynamic Pol III identity during development

The isolation and identification of Pol III_A_ and III_B_ in mouse myeloma tumor cells materialized in a context in which both RPC7 subunits were co-expressed and could be directly compared (Schwartz et al., 1974). However, examination of human RPC7α and RPC7β levels across diverse tissues and cell lines has revealed a spatiotemporal regulation of Pol III subunit composition, such that RPC7α is transiently expressed in early developmental windows and replaced with RPC7β, which becomes constitutively expressed in differentiated tissues (Haurie et al., 2010; Wong et al., 2011; Lund et al., 2017) (Figure 2B). In mice, *Polr3g* and *Polr3gl* mRNA levels are low at the earliest 2- and 4-cell stages of mouse development, followed by rapid upregulation of *Polr3g* at the 8-cell stage and peaking at the blastula stage (Wang et al., 2020). In humans, *POLR3G* expression is regulated by pluripotency factors OCT4 and NANOG, with reported binding sites upstream and downstream of the *POLR3G* transcription start site (Wong et al., 2011). Reduction of *POLR3G* expression induces loss of pluripotency and promotes differentiation of human embryonic stem cells (hESCs), suggesting an important role in hESC maintenance. *POLR3G* expression is rapidly downregulated during either spontaneous embryonic body differentiation or retinoic-acid-induced differentiation of hESC cells (Lund et al., 2017). *POLR3GL* expression and RPC7β protein levels are conversely upregulated during hESC differentiation and remain abundant in most tissues, suggesting RPC7β, the putative ancestral RPC7 subunit, is the predominant form of Pol III across non-stem cell contexts. *POLR3GL* expression peaks in skeletal and heart muscle, implying a potentially important role for RPC7β and Pol III levels in these tissues.

During mouse development, functional null mutations in *Polr3g* cause embryonic lethality between stages E3.5 and E6.5, whereas analogous disruption of *Polr3gl* produces postnatal growth defects resulting in premature death 3 weeks after birth (Wang et al., 2020). While the differential effects of *Polr3g* and *Polr3gl* mutants point to differences in spatiotemporal availability, heterozygous mutations in the opposite subunit further aggravate the lethal phenotypes in *Polr3g* ^*fn/fn*^ *and Polr3gl* ^*fn/fn*^ *,* demonstrating at least partial compensation by the opposite RPC7 subunit during development. Indeed, *POLR3G* levels are significantly upregulated in tissues of *Polr3gl* ^*fn/fn*^ mice, implying a potential feedback and compensation mechanism when POLR3GL is lost (Wang et al., 2020). However, despite compensatory increases in *POLR3G*, *Polr3gl* ^*fn/fn*^ mice are characterized by abnormal size and cerebellar function defects, perhaps revealing an important role for *POLR3GL* in these and other contexts. As we will discuss below, evidence suggests that subunit RPC7α expands and enhances the Pol III transcriptome, perhaps explaining how *POLR3G* overexpression sufficiently compensates for loss of *POLR3GL*, albeit leading to nuanced developmental abnormalities and premature death. On the other hand, *POLR3GL* can compensate for loss of *POLR3G* in cellular models of early development (Wang et al., 2020), but whether *Polr3g* ^*fn/fn*^ mice are able to develop normally, if transiently rescued during early stages of embryonic development, remains unaddressed.

## Regulation of *POLR3G* and *POLR3GL* and dynamic Pol III transcription

In addition to the spatiotemporal regulation and dynamic replacement of subunit RPC7α with RPC7β during early development, *POLR3G* expression re-emerges in proliferative and transformed cells, in line with the discovery of both Pol III_A_ and III_B_ in mouse myeloma tumor cells (Schwartz et al., 1974; Haurie et al., 2010). In these contexts, upregulation of *POLR3G* is likely driven by the transcription factor and oncogene, MYC, which directly targets the promoter region of *POLR3G* but is not observed at *POLR3GL* (Renaud et al., 2014). These findings gave rise to a model, proposed by Nouria Hernandez and colleagues, in which the duplication of RPC7 evolved to facilitate dynamic increases in Pol III levels in response to growth stimuli. In this model, *POLR3GL* functions to provide a constitutive baseline for Pol III availability, and Pol III activity and target specificity are driven solely by the level of RPC7 availability, enhanced by MYC-mediated *POLR3G* upregulation (Renaud et al., 2014).

However, above and beyond increasing RPC7 subunit availability, recent studies suggest subunit RPC7α functionally enhances Pol III transcription more significantly than *POLR3GL* and RPC7β. Overexpression of *Polr3g* increases tRNA abundance more robustly compared to *Polr3gl* overexpression in *X. tropicalis* embryos, for example, and several tRNA species are paradoxically downregulated in cells with elevated *Polr3gl* expression (McQueen et al., 2019). In human cell lines, differentiation-induced loss of *POLR3G* expression is accompanied by significant downregulation of Pol III activity at a subset of Pol III-transcribed genes, further evidence that RPC7α availability enhances Pol III transcription (Van Bortle et al., 2022). In particular, small NF90-associated RNA (snaR), a small ncRNA expressed in testis, transformed cell lines, and tumors, selectively loses Pol III occupancy and transcription. This phenomenon is also observed at *BCYRN1*, a primate and tissue-specific Pol III-transcribed gene, and a subrepertoire of tRNA genes, whereas the rest of the Pol III transcriptome remains relatively unperturbed by diminished RPC7α levels in immortalized THP-1 cells (Van Bortle et al., 2022). However, *POLR3G* knockout in MDA-MB-231 triple negative breast cancer (TNBC) cells does not lead to significant changes in the steady-state levels of BC200, the small ncRNA encoded by *BCYRN1*, suggesting RPC7α enhancement may be context-dependent or may depend on additional factors (Lautré et al., 2022). These results indicate a nuanced role for RPC7α in shaping Pol III transcription with implications for the dynamic Pol III signatures observed during development and in human disease contexts.

## Pol III identity and cancer

The re-emergence of RPC7α in transformed cell lines is concordant with evidence that *POLR3G* expression increases in a variety of cancers. *POLR3G* is selectively upregulated in prostate cancer, for example, in contrast to *POLR3GL* and the large Pol III subunit *POLR3A*, which remain unchanged compared to matched normal cells (Petrie et al., 2019). In breast cancer, *POLR3G* is specifically overexpressed in basal-like tumors, which are often associated with unfavorable prognosis (Lautré et al., 2022). Functional knockout of *POLR3G* in MDA-MB-231, a TNBC cell line frequently classified as a basal-like breast cancer subtype, reduces anchorage-independent growth and invasive capacities *in vitro*. Mammary tumor growth and metastatic dissemination are significantly reduced in orthotopically xenografted knockout cells, demonstrating that the availability of RPC7α plays an important role in tumorigenesis *in vivo* (Lautré et al., 2022). *POLR3G* knockout did not disrupt the proliferation rate of MDA-MB-231 cells, however, suggesting RPC7α levels and downstream Pol III dynamics may confer selective advantages for invasion and metastasis rather than proliferation, or that additional factors or events are important for understanding the contribution of RPC7α to cell growth.

Notably, *POLR3G* upregulation has been linked with poor survival outcomes across a variety of cancers, including in patients with lung adenocarcinoma (Sun et al., 2022), hepatocellular carcinoma (Dai et al., 2021), transitional cell carcinoma (Liu et al., 2020), multiple myeloma (Yang et al., 2021), and other forms of cancer (Van Bortle et al., 2022). In fact, pan-cancer comparison of survival signatures across all cancer subtypes reveals that *POLR3G* expression is the most significant feature and predictor of poor survival outcomes compared with all other Pol III subunits (Figure 2A) (Smith and Sheltzer, 2022). Genes encoding Pol III subunits RPC3 (*POLR3C*) and RPC9 (*CRCP*), both of which structurally and functionally intersect RPC7α, have similarly strong signatures. In contrast, *POLR3GL* expression is the only signature strongly associated with favorable outcomes when analyzed across all cancer types (Figure 2A). Taken together, these findings implicate *POLR3G* in disease progression and identify Pol III subunit RPC7α as a promising molecular target in cancer.

The contrasting relationship between *POLR3G* and *POLR3GL* overexpression and cancer outcomes is, on its face, incongruous with a model in which both RPC7 subunits are functionally identical and raises questions about the regulation and function of Pol III identity in disease. The regulatory mechanisms underlying *POLR3G* overexpression are likely to include master transcription factors that are often subverted during oncogenesis (Reddy et al., 2021), both connecting and, likely to some degree, confounding *POLR3G* expression with dysregulated regulatory programs. *POLR3G* embryonic regulators OCT4 and NANOG, for example, are markers of cancer stem cells and play important roles in tumor-initiating cells (Wang and Herlyn, 2015). In PC-3 prostate cancer cells, *POLR3G* is directly regulated by NANOG, and reciprocally influences the levels of NANOG through the expression of DR2 Alu elements (Petrie et al., 2019). Specifically, RPC7α inefficiently transcribes DR2 Alu elements that otherwise produce small ncRNAs capable of disrupting NANOG mRNA levels. Loss of *POLR3G* expression permits Pol II occupancy, efficient DR2 Alu expression, and targeted downregulation of *NANOG* (Petrie et al., 2019). Regulation of *POLR3G* may also intersect specific miRNAs, such as miR-1305, a microRNA (miRNA) implicated in the posttranscriptional regulation of *POLR3G* during early development (Jin et al., 2016). miR-1305 reportedly restricts cancer progression across multiple subtypes, though the connection between miR-1305 and *POLR3G* expression in cancer remains poorly studied.

Beyond the embryonic transcription factors OCT4, NANOG, and putative miR-1305 interference, *POLR3G* expression is also regulated by MYC and sensitive to MYC disruption in cancers featuring MYC upregulation, including colon carcinoma and acute myeloid leukemia cell lines (Renaud et al., 2014; Van Bortle et al., 2022). Upregulation of Pol III activity is a hallmark of cancer, and several studies have linked MYC to Pol III-transcribed genes, Pol III transcription factors, and increased Pol III activity (Gomez-Roman et al., 2003; Goodfellow et al., 2006; Kenneth et al., 2007; Campbell and White, 2014). Knockdown of MYC disrupts both the expression of *POLR3G* and the levels of snaR-A ncRNA, linking MYC regulation with the RPC7α-sensitive transcriptome (Van Bortle et al., 2022). In addition to snaR ncRNA, both *BCYRN1* and specific RPC7α-enhanced tRNA genes are also sensitive to MYC disruption (Hu and Lu, 2015; Gerber et al., 2020), likely intersecting Pol III identity and transcription. Though *POLR3G* expression broadly correlates with MYC levels across all cancer types, whether and to what degree MYC activity overlaps OCT4, NANOG, and potentially other context-specific master transcription factors remain important questions.

## Disease mutations in RPC7β

In addition to connections between RPC7 subunit composition and cancer, specific mutation events also link RPC7 to rare conditions and developmental disorders. Genetic mutations in several Pol III subunits result in a variety but overlapping set of disease states, including autoimmune conditions initiated by genetic alterations of *POLR3A* in cancer (Joseph et al., 2014), immunodeficiencies caused by mutation events in *POLR3A*, *POLR3C*, *POLR3E*, and *POLR3F* (Carter-Timofte et al., 2019), and hypomyelinating leukodystrophy and related neurodegenerative conditions caused by distinct mutations in *POLR3A*, *POLR3B*, *POLR1C*, and *POLR3K* (Lata et al., 2021; Moir et al., 2021). Most leukodystrophy-causing mutations result in partial loss-of-function and reduced Pol III activity, perturbing levels of BC200 and specific tRNAs (Yeganeh and Hernandez, 2020). Biallelic alterations in *POLR3A* also result in Wiedemann-Rautenstrauch Syndrome (WRS), a variant of neonatal progeroid syndrome characterized by growth restriction, macrocephaly, and lipodystrophy (Wambach et al., 2018). A homozygous non-sense mutation in *POLR3GL*, shown to result in premature termination and significant loss of *POLR3GL* mRNA levels, has been linked with similar clinical features, identifying RPC7β disruption as an additional causal factor in WRS (Beauregard-Lacroix et al., 2020). Though it wasn’t reported, the observed genetic disruption of *POLR3GL* in humans would theoretically require autocompensation by *POLR3G*, as reported in *Polr3gl* ^*fn/fn*^ mice (Wang et al., 2020). Splice acceptor site mutations in *POLR3GL*, which ostensibly do not eliminate *POLR3GL* expression but instead produce abnormal forms of subunit RPC7β, also cause endosteal hyperostosis, oligodontia, and mild neurological features (Terhal et al., 2020). Altogether, the clinical growth and intellectual abnormalities reported are in line with *Polr3gl* ^*fn/fn*^ phenotypes and would again suggest an important role for *POLR3GL*, and perhaps the absence of *POLR3G*, in the brain and other tissues (Figure 2B). To date, no analogous studies have identified significant genetic alterations in *POLR3G*, possibly due to the essentiality of RPC7α during early development and, potentially, other contexts.

## ML-60218, an RPC7α-specific inhibitor?

The Pol III transcriptome is integral in basic cellular processes that support and promote growth, and thus inhibiting Pol III in cancer may represent a promising therapeutic strategy. However, since the discovery of the three eukaryotic RNA polymerase enzymes in 1969 (Roeder and Rutter, 1969) and early characterizations of distinct α-amanitin sensitivities (Weinmann and Roeder, 1974), a minimal number of Pol III inhibitors have been identified—in contrast to the growing collection of multi-approach Pol II inhibitors (Martin et al., 2020). Among the shortlist of Pol III inhibitors are antibiotic and antifungal compounds, including tagetitoxin, a bacterial phytotoxin that naturally inhibits chloroplast RNA polymerase and also preferentially inhibits human Pol III *in vitro* (Steinberg et al., 1990), and UK-118005, a broad-spectrum antifungal compound that rapidly inhibits tRNA synthesis in *S. cerevisiae* (Wu et al., 2003). A small molecule analog of UK-118005, ML-60218, was discovered as a potent inhibitor of Pol III derived from human cells (Wu et al., 2003).

While ML-60218 may broadly inhibit Pol III transcription, in humans, the drug is intriguingly ineffective in contexts with RPC7β that are absent RPC7α. For example, ML-60218 only modestly perturbs Pol III transcription in preadipocytes and is entirely ineffective in terminally differentiated cells (Chen et al., 2018). ML-60218 is otherwise effective at inhibiting Pol III transcription in contexts with high *POLR3G* expression, including breast and prostate cancer cell lines (Nabet et al., 2017; Petrie et al., 2019). Genomic profiling of small RNA levels in response to ML-60218 in THP-1, an acute myeloid leukemia cell line, identifies a specific subrepertoire of Pol III-transcribed genes that are significantly reduced in response to drug exposure, including snaR ncRNA and other putatively *POLR3G*-sensitive small ncRNAs, whereas most Pol III-transcribed genes are insensitive (Van Bortle et al., 2022). ML-60218 selectively reduces RPC7α while increasing RPC7β occupancy in THP-1, consistent with evidence that ML-60218 reshapes Pol III complex identity in favor of RPC7β in PC-3 cells and altogether supporting a model in which ML-60218 selectively disrupts Pol III with RPC7α incorporated (Petrie et al., 2019; Van Bortle et al., 2022). ML-60218 exposure stimulates expression of markers indicative of neuroendocrine differentiation in PC-3 cells (Petrie et al., 2019), and enhances adipocyte differentiation, but otherwise interferes with osteoblast differentiation (Phillips et al., 2022), suggesting a potentially complex interplay of Pol III identity and developmental context influences the observed effects of ML-60218.

Though the precise mechanism of selective Pol III inhibition remains unaddressed, structural prediction of ML-60218 binding identifies a presumed target, Gly-1045 of the large Pol III subunit RPC1 (Kessler and Maraia, 2021). The location of ML-60218 docking, between the bridge helix and trigger loop helix, implies an inhibitory mechanism that targets the core site of transcription, similar in nature to α-amanitin. Drawing from recent structural reports of the Pol III complex, predicted differences in RPC7α interactions over the clamp domain, which are expected to bind more strongly than RPC7β (Girbig et al., 2021), may suggest a tighter structural configuration more sensitive to ML-60218 insertion. Alternate models intersect the potential autoinhibitory role of the RPC7 C-terminus at the core site of transcription (Kessler and Maraia, 2021). The bias of ML-60218 in disrupting RPC7α, the Pol III subunit upregulated in cancer and most strongly associated with unfavorable patient outcomes, points the way for future small molecule inhibitors and potential cancer drug candidates.

## Conclusion

Pol III activity is a core, underlying engine fundamental to the growth and proliferation of both normal and cancer cells. The regulatory mechanisms driving increased Pol III and altered transcription patterns during tumorigenesis likely intersect most cancer subtypes and represent an important avenue for understanding cancer initiation and progression. Advances in Cryo-EM resolution and recent applications to Pol III have uncovered new insight into the structure, function, and regulation of the Pol III complex (Ramsay et al., 2020; Li et al., 2021), and recent biochemical and genomic studies have captured important changes in Pol III transcription in both normal and cancer contexts (Van Bortle et al., 2017; McQueen et al., 2019; Van Bortle et al., 2022).

In this review, we have focused on just one putative regulatory mechanism: Pol III subunit composition. The re-emergence of *POLR3G* expression in cancer and statistical relationship with unfavorable outcomes, in stark contrast to *POLR3GL*, is undeniable (Figure 2A). Nevertheless, the varying levels of distinction observed between RPC7α and RPC7β at molecular and phenotypic levels have clouded our current understanding of whether the paralogous RPC7 subunits are functionally divergent or simply redundant. When considering the current body of work, it is likely that the differences are subtle yet consequential. Differences are implied from structural analysis of RPC7 protein-protein interactions, including distinct properties predicted over the clamp domain (Girbig et al., 2021). Differences are observed in Pol III transcription in contexts overexpressing *POLR3G* or *POLR3GL* (McQueen et al., 2019), and during differentiation-associated loss of *POLR3G* or re-emergence of *POLR3G* in cancer (Van Bortle et al., 2022). Differences are also observed in the long-term viability and development of mice and humans with loss-of-function genetic mutations in *POLR3GL* despite, in mouse models, evidence of *POLR3G* autocompensation (Beauregard-Lacroix et al., 2020; Terhal et al., 2020; Wang et al., 2020). Fully understanding the contributions of RPC7α and RPC7β to development and disease outcomes will require genomic and other approaches sensitive enough to identify subtle changes in Pol III activity, and secondary functions of both RPC7α and RPC7β must also be considered.

The growing evidence of Pol III identity-driven differences in transcription and development is met with added questions about the regulation, mechanism, and differential function of RPC7α and RPC7β. The regulation of *POLR3G* by OCT4, NANOG, MYC, and potentially other master transcription factors, may also extend to forms of post-transcriptional regulation and alternative isoform usage. Comparatively little is currently known about the regulation of *POLR3GL* expression, despite evidence of tissue-specific increases in *POLR3GL* mRNA levels. These questions are of particular interest following evidence of autocompensation between POLR3G and POLR3GL (Wang et al., 2020), implying either unmapped feedback mechanisms or selective pressures during early development. The mechanism of RPC7α-enhanced transcription, either through modulation of MAF1 repression, differential levels of autoinhibition, or other means, remains a major question for the field. Finally, beyond simply distinguishing RPC7α and RPC7β, whether the unusual co-expression and concurrent availability of RPC7α and RPC7β in cancer presents a special scenario of biological significance remains an important matter for future research.
